# Identification of UBE2C as hub gene in driving prostate cancer by integrated bioinformatics analysis

**DOI:** 10.1371/journal.pone.0247827

**Published:** 2021-02-25

**Authors:** Yan Wang, Jili Wang, Qiusu Tang, Guoping Ren

**Affiliations:** 1 Department of Pathology, The First Affiliated Hospital, Zhejiang University School of Medicine, Hangzhou, China; 2 Department of Pathology and Pathophysiology, Zhejiang University School of Medicine, Hangzhou, China; University of Nebraska Medical Center, UNITED STATES

## Abstract

**Background:**

The aim of this study was to identify novel genes in promoting primary prostate cancer (PCa) progression and to explore its role in the prognosis of prostate cancer.

**Methods:**

Four microarray datasets containing primary prostate cancer samples and benign prostate samples were downloaded from Gene Expression Omnibus (GEO), then differentially expressed genes (DEGs) were identified by R software (version 3.6.2). Gene ontology (GO) and Kyoto Encyclopedia of Genes and Genomes (KEGG) were performed to identify the function of DEGs. Using STRING and Cytoscape (version 3.7.1), we constructed a protein-protein interaction (PPI) network and identified the hub gene of prostate cancer. Clinical data on GSE70770 and TCGA was collected to show the role of hub gene in prostate cancer progression. The correlations between hub gene and clinical parameters were also indicated by cox regression analysis. Gene Set Enrichment Analysis (GSEA) was performed to highlight the function of Ubiquitin-conjugating enzyme complex (UBE2C) in prostate cancer.

**Results:**

243 upregulated genes and 298 downregulated genes that changed in at least two microarrays have been identified. GO and KEGG analysis indicated significant changes in the oxidation-reduction process, angiogenesis, TGF-beta signaling pathway. UBE2C, PDZ-binding kinase (PBK), cyclin B1 (CCNB1), Cyclin-dependent kinase inhibitor 3 (CDKN3), topoisomerase II alpha (TOP2A), Aurora kinase A (AURKA) and MKI67 were identified as the candidate hub genes, which were all correlated with prostate cancer patient’ disease-free survival in TCGA. In fact, only UBE2C was highly expressed in prostate cancer when compared with benign prostate tissue in TCGA and the expression of UBE2C was also in parallel with the Gleason score of prostate cancer. Cox regression analysis has indicated UBE2C could function as the independent prognostic factor of prostate cancer. GSEA showed UBE2C had played an important role in the pathway of prostate cancer, such as NOTCH signaling pathway, WNT-β-catenin signaling pathway.

**Conclusions:**

UBE2C was pivotal for the progression of prostate cancer and the level of UBE2C was important to predict the prognosis of patients.

## Introduction

Prostate cancer has become the most universal diagnosed cancer among American men, which accounted for 20% of newly diagnosed cancers [1,2]. To our surprise, although the survival probability of prostate cancer was the highest, the proleptic mortality of prostate carcinoma accounted for 10% among the whole cancer death cases [1]. Among American men, prostate cancer remained the third-leading reason for cancer death. Based on the cancer statistics in China, we have concluded that prostate cancer has become the most popular cancer of the genitourinary system of men [2]. In detail, from 2000 to 2011, the occurrence of prostate cancer has increased year by year. Prostate cancer has become a threat to long-term health of patients and has worsened the living conditions. Unfortunately, in China, the mortality of prostate cancer has been rising year by year too. It was essential for clinicians to diagnose and to treat diverse kinds of prostate cancer correctly and appropriately. For localized prostate cancer, surgery and radiation have become the first choice, although it could bring some adverse effects to patients, such as frequent urination, hematuria and sexual dysfunction that maybe pull down life quality [3]. Androgen deprivation therapy(ADT) combined with radiotherapy was superior to other therapies for high risk prostate cancer patients [4]. Although ADT was recognized as standard therapy for diverse prostate cancer, chemotherapy has also been recommended for metastatic prostate cancer. In terms of the screening and diagnosis, prostate-specific antigen (PSA) has been widely performed to monitor and diagnose prostate cancer. While due to PSA’s poor specificity, early PSA test, could lead to overdiagnosis and overtreatment of prostate cancer [5]. Previous studies have proved high-sequencing could function as a novel way to identify biomarkers in various cancers. Fang et al. have compared stroma surrounding invasive prostate tumors and matched normal stroma, in order to identify a potential target for prostate cancer [6]. Comparing luminal cells, basal cells and epithelial cells from hormone-naive and castration-resistant mice, L6YD was identified as a marker castration-resistant prostate cancer [7]. Thus, this article aimed to identify the hub genes of prostate carcinoma by bioinformatics analysis.

In this study, four databases were downloaded from GEO, which included benign prostate gland and primary prostate cancer tissue. Using limma and affy package of R software, DEGs were identified. Furthermore, GO and KEGG pathway analysis were performed by The Database for Annotation, Visualization, and Integrated Discovery (DAVID) [8] (http://www.david.niaid.nih.gov). Then, PPI and modules analysis was performed by STRING [9] (https://string-db.org/), metascape [10] (http://metascape.org/gp/index.html) and Cytoscape [11,12]. Survival analysis was performed to screen and validate the hub gene of prostate cancer. In brief, UBE2C, highly expressed in prostate cancer tissue, was identified as the real hub gene. Through GSEA [13] and univariate and multivariate analysis, the role of UBE2C in prostate cancer was explored completely.

## Materials and methods

### Microarray data

GSE104749 [14], GSE3325 [15], GSE69223 [16], GSE46602 [17], four prostate cancer gene expression profiles were downloaded from GEO, which were from the same GPL platform (GPL570). The criteria of inclusion GEO gene expression profiles were shown in S1 Fig. GSE3325 contained 7 localized prostate cancer tissues and 6 benign prostate tissues. GSE46602 contained 36 prostate tumor samples and 14 normal prostate gland samples. GSE69223 included 15 cancer tissues and 15 corresponding noncancerous tissue samples. GSE104749 contained 4 prostate cancer samples and 4 benign prostate gland samples (Table 1). The clinicopathological features of 206 primary prostate cancer patients in the GSE70770 were collected for further analysis.

**Table 1:** Characteristics of the microarray datasets.

Accession/ID	PMID	Experimental group	Control group
GSE3325	16286247	Prostate cancer tissue samples (7)	Benign prostate tissue samples (6)
GSE46602	26522007	Prostate cancer tissue samples (36)	Normal prostate gland samples (14)
GSE69223	26623558	Prostate cancer tissue samples (15)	Noncancerous tissue samples (15)
GSE104749	29285211	Prostate cancer tissue samples (4)	Benign prostate tissue samples (4)

### Identification of DEGs

**Fig 1:**
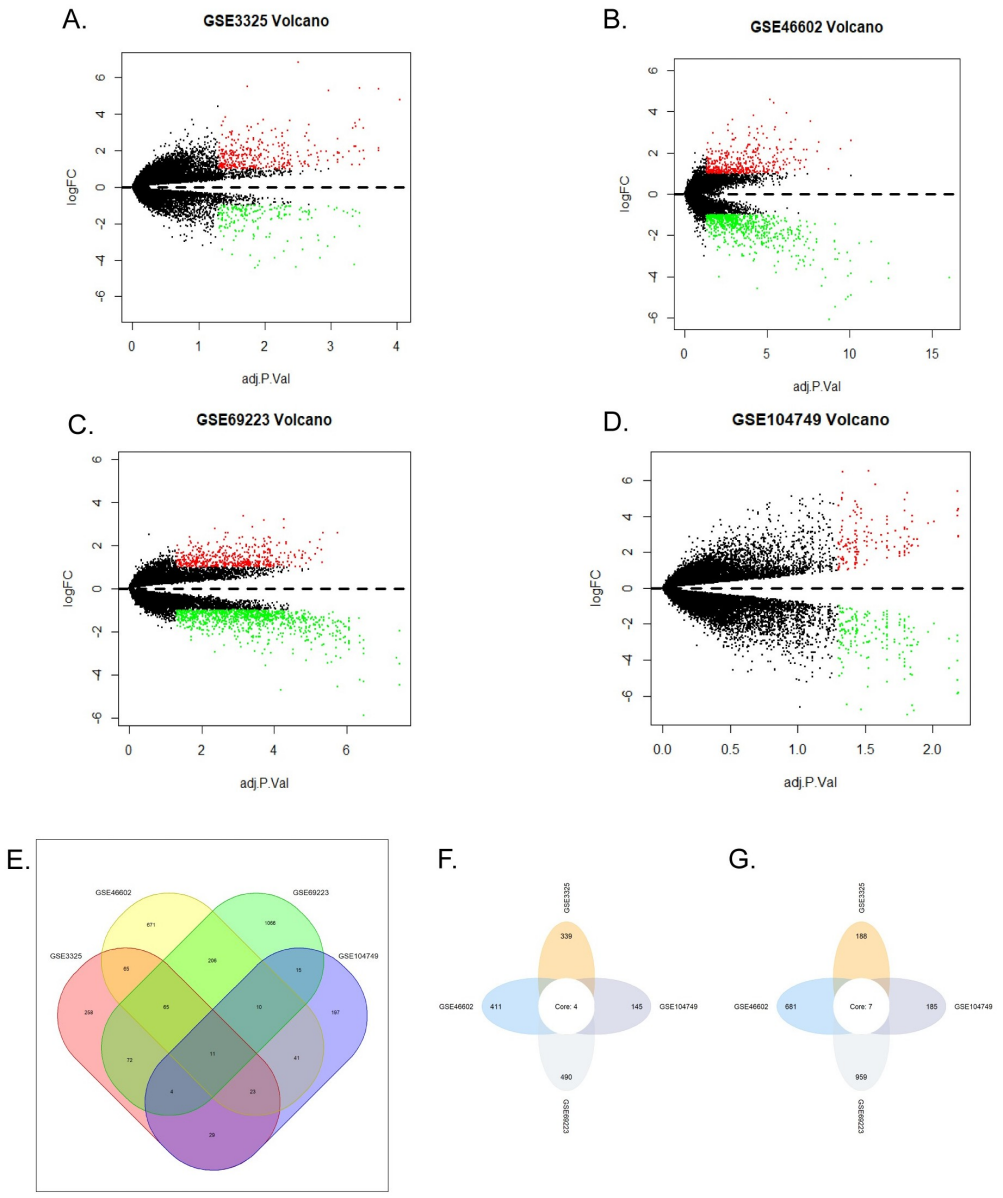
DEGs in GSE104749, GSE3325, GSE69223, GSE46602. (A-D) Volcano plots of all genes in four GEO databases. Red plots represented upregulated genes, green represented downregulated genes, the black plots represented the genes with no significant changes in expression. (A) GSE3325. (B) GSE46602. (C) GSE69223. (D) GSE104749. (E) Venn diagram of DEGs in four databases. The green, yellow, red, purple colors represented the DEGs of GSE46602, GSE69223, GSE104749, GSE3325, respectively. The number on the crossing area indicated the cross DEGs that were owned by different databases. (F) Upregulated genes co-expressed in the four databases. (G) Downregulated genes co-expressed in the four databases.

GSE3325, GSE46602, GSE69223 and GSE104749 gene expression profiles have been normalized and transformed into suitable matrix using Robust Multichip Average (RMA) included in affy package of R software (version 3.6.1) consisting of background adjustment, quantile normalization and summarization pre-processing [6,18]. Limma package was used to identify DEGs between primary prostate cancer and noncancerous prostate samples. The criteria of DEGs were adjusted p value < 0.05 and |log2 (fold change) | > 1. Venn diagram was used to find DEGs appearing in at least two databases (Figs 1 and S2).

### GO and KEGG pathway analysis of the DEGs

**Fig 2:**
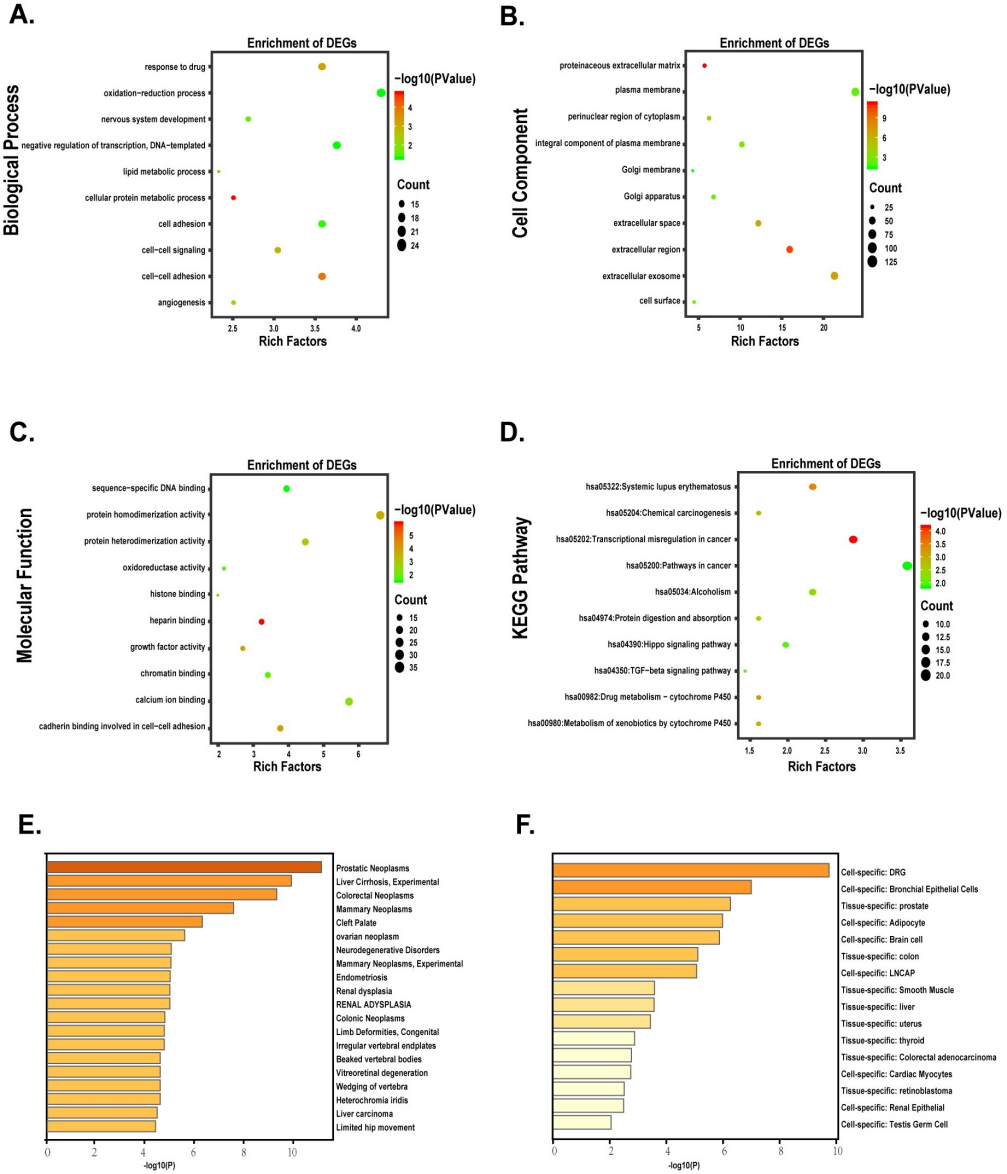
Functional analysis of DEGs. (A-D) The bubble plots indicated the results of function enrichment analysis in DEGs. (A) Biological process. (B) Cell component. (C) Molecular function. (D) KEGG pathways. (E-F) Different expression genes enriched in the specific tissues and cell lines.

To further explore the function of DEGs, GO [19] and KEGG [20] analysis of DEGs was performed by DAVID. We downloaded the results and performed further analysis by the R software. The criterion of function analysis was p-value cutoff <0.05. Using metascape, DEGs were studied for further (Fig 2).

### Construction and analysis of PPI network

**Fig 3:**
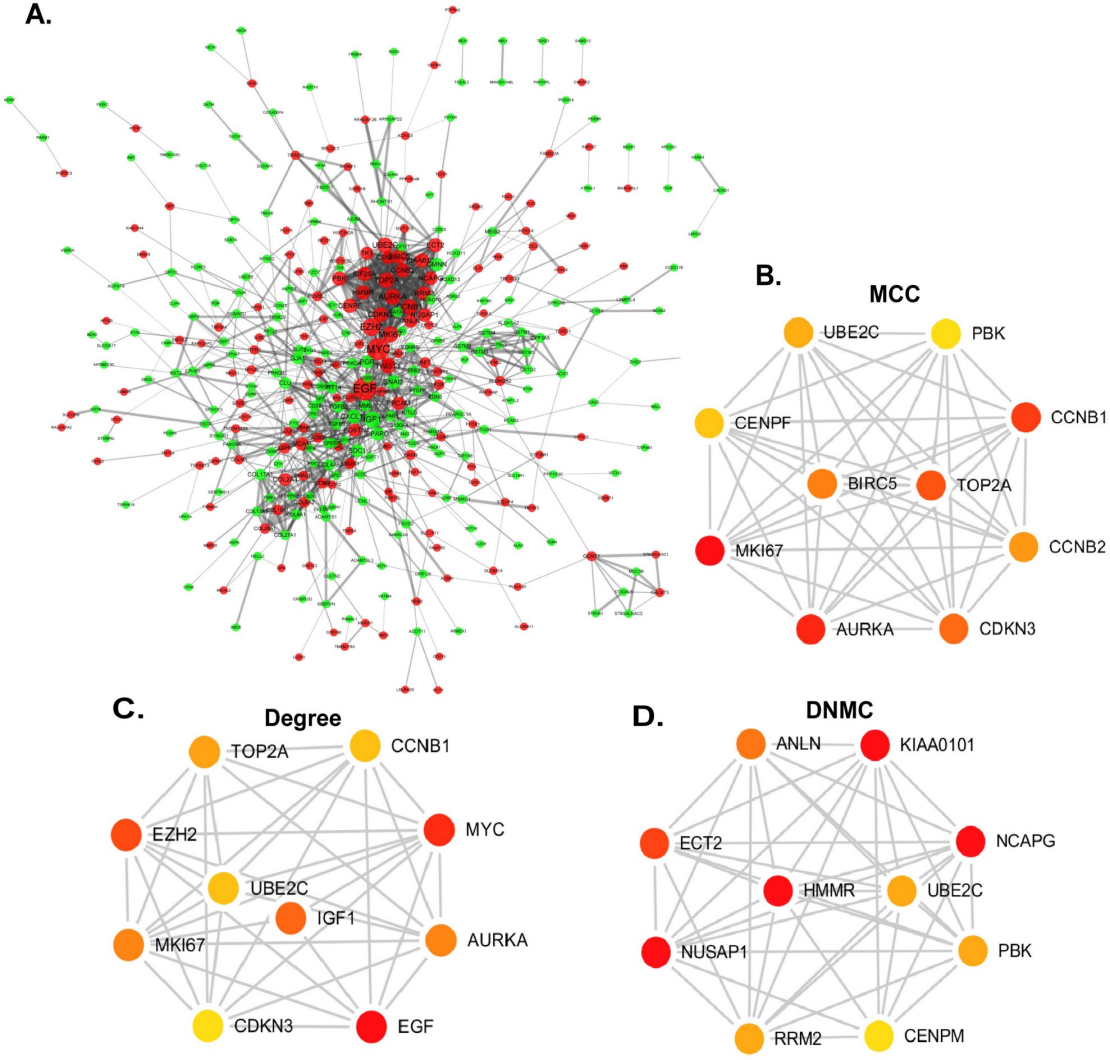
Construction of PPI network and identification of candidate hub genes. (A) PPI network. Red nodes represented upregulated genes while green represented downregulated genes. (B-D) Three different methods of cytoHubba were preformed to identify the hub genes. The top ten nodes are shown with a color scheme from red (highly important) to green (important). (B) Top ten genes in the PPI network were calculated by MCC. (C) Top ten genes in the PPI network were selected by Degree as candidates. (D) Top ten genes in the PPI network were selected by DNMC.

Online website STRING and cytoscape were used to build the PPI network and analyzed the functional pathways (Fig 3). Cytoscape functioned as a network graph, with differential expressed molecules represented as nodes and intermolecular interactions represented as links, that is, edges, between nodes [11]. In this study, the combined score represented intermolecular interactions and the protein pairs with combined score > 0.4 were selected to construct the PPI network [21]. Nodes were drawn in different sizes and colors, which represented the node degree and the regulation (up or down), respectively [18]. The hub genes were calculated by different methods with cytohubba [22]. The cytohubba is a plugin of cytoscape. It was widely used and essential for us to explore the most important node in diverse biological networks. CytoHubba included 11 topological analysis methods, while in this study we used Degree, Density of Maximum Neighborhood Component (DNMC), Maximal Clique Centrality (MCC) to identify candidate hub gene of prostate cancer.

### Survival analysis and expression validation of candidate hub genes

**Fig 4:**
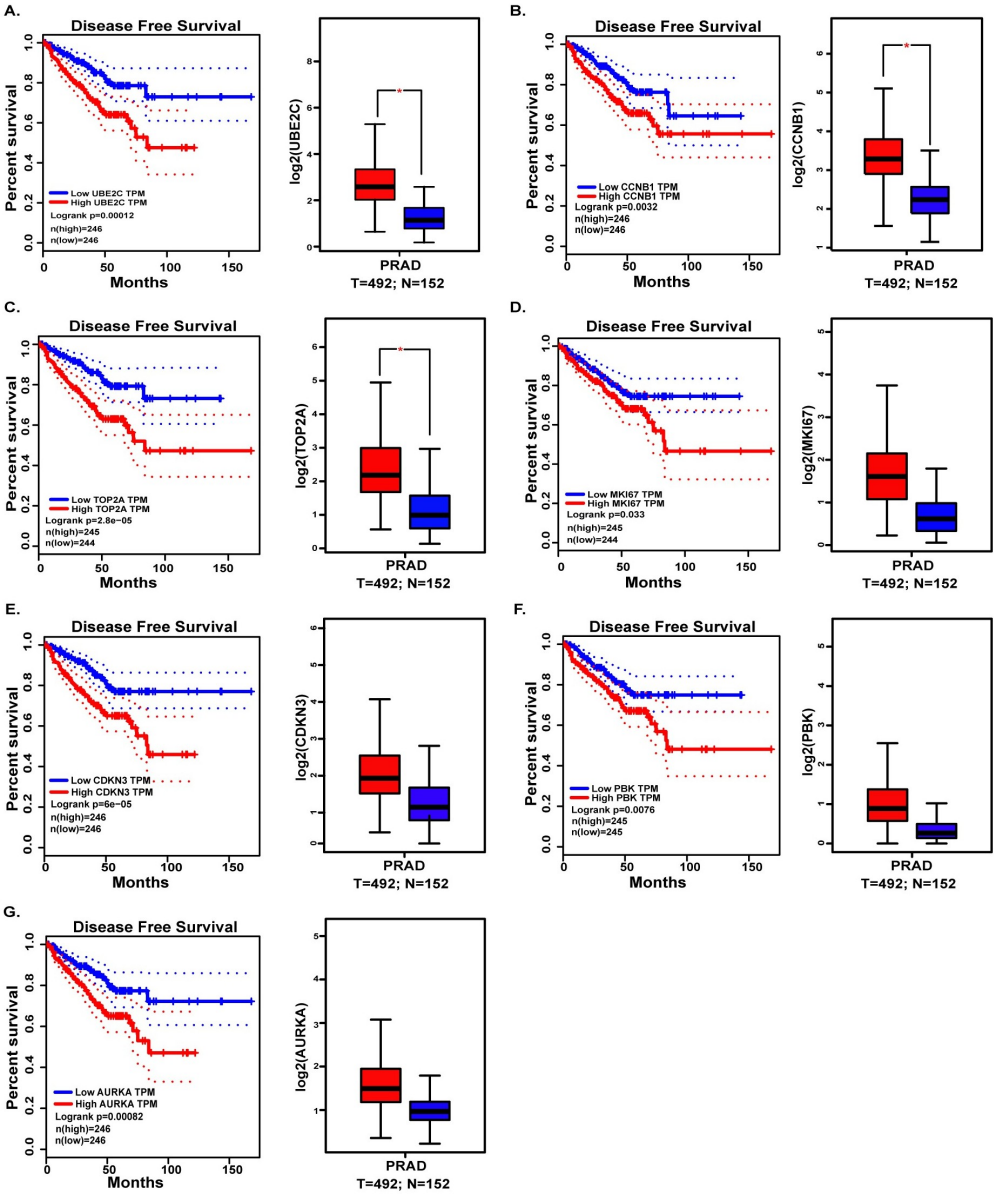
Validation the expression and disease-free survival (DFS) of candidate hub genes in TCGA. (A) UBE2C. (B) CCNB1. (C) TOP2A. (D) MKI67. (E) CDKN3. (F) PBK. (G) AURKA.

To further figure out the real hub gene from candidate hub genes of prostate cancer, Gene Expression Profiling Interactive Analysis (GEPIA, http://gepia.cancer-pku.cn/) [23], an online website based on The Cancer Genome Atlas [24] (TCGA) and the Genotype-Tissue Expression (GTEx) databases [25], was not only performed to valid the expression of candidate hub genes between prostate cancer and benign prostate tissue, but also used to validate the correlations between disease-free survival and expression level of candidate hub genes (Fig 4).

### Clinical parameters analysis

**Fig 5:**
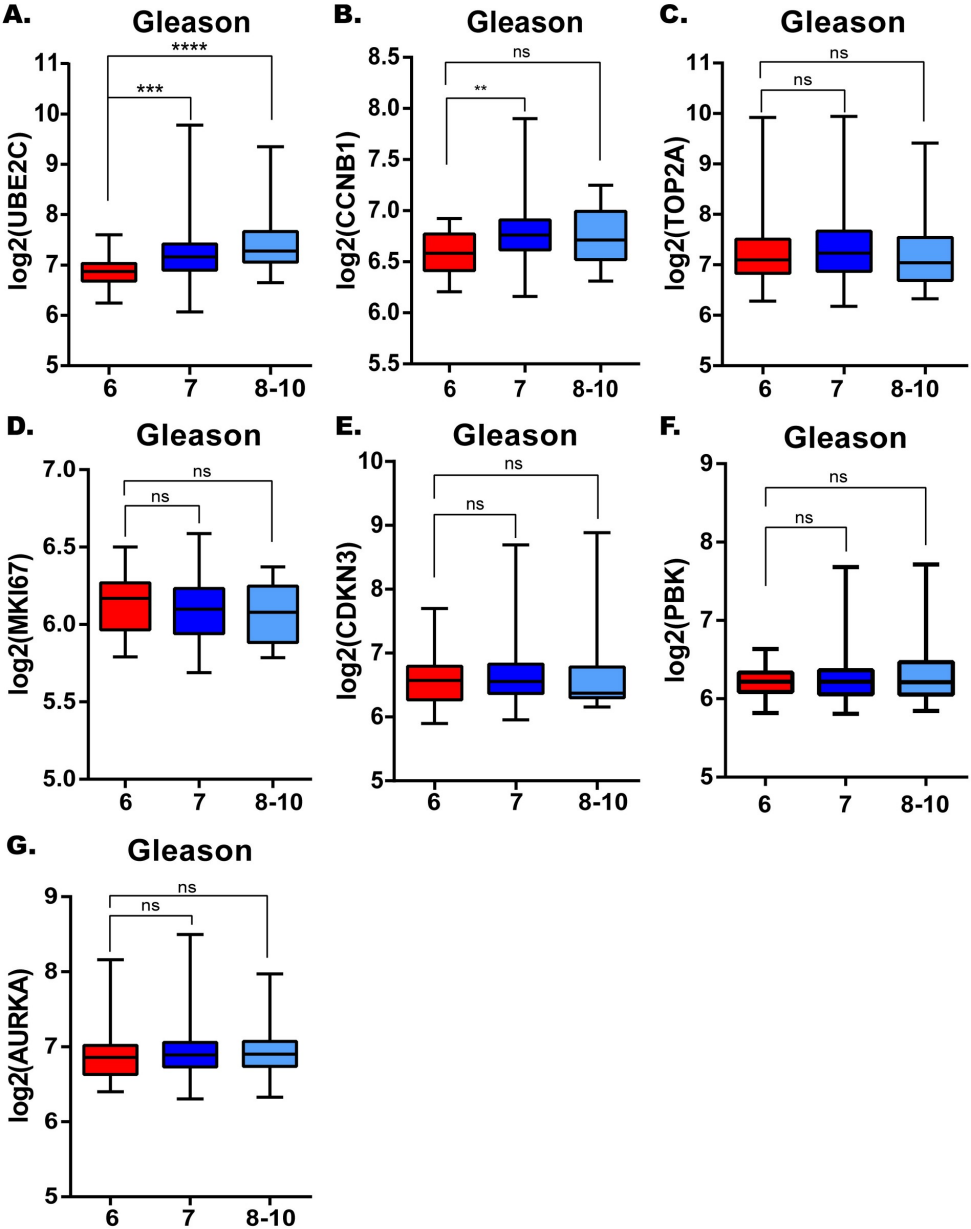
The correlations between candidate hub genes and Gleason score. UBE2C. (B) CCNB1. (C) TOP2A. (D) MKI67. (E) CDKN3. (F) PBK. (G) AURKA. *: p<0.05; **: p<0.01; ***: p<0.001; ****: p<0.0001.

**Fig 6:**
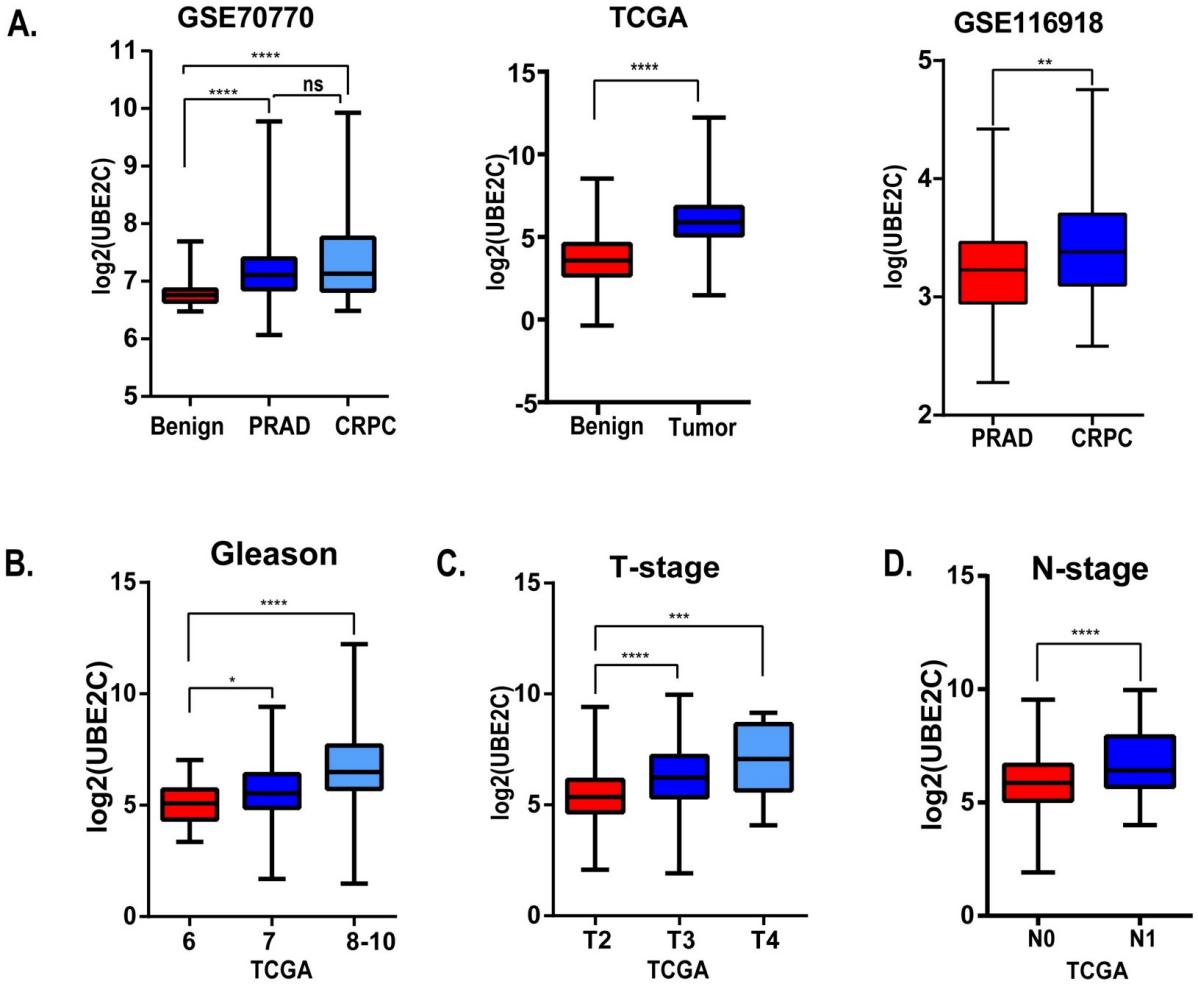
The correlations between UBE2C and clinical parameters. (A) The expression level of UBE2C of benign prostate tissue, prostate cancer and castration-resistant prostate cancer in GSE70770 (left), TCGA (middle) and GSE116918 (right). (B) The relationship between Gleason score and UBE2C in TCGA. (C) The relationship between T-stage and UBE2C in TCGA. (D) The relationship between N-stage and UBE2C in TCGA. *: p<0.05; **: p<0.01; ***: p<0.001; ****: p<0.0001.

**Fig 7:**
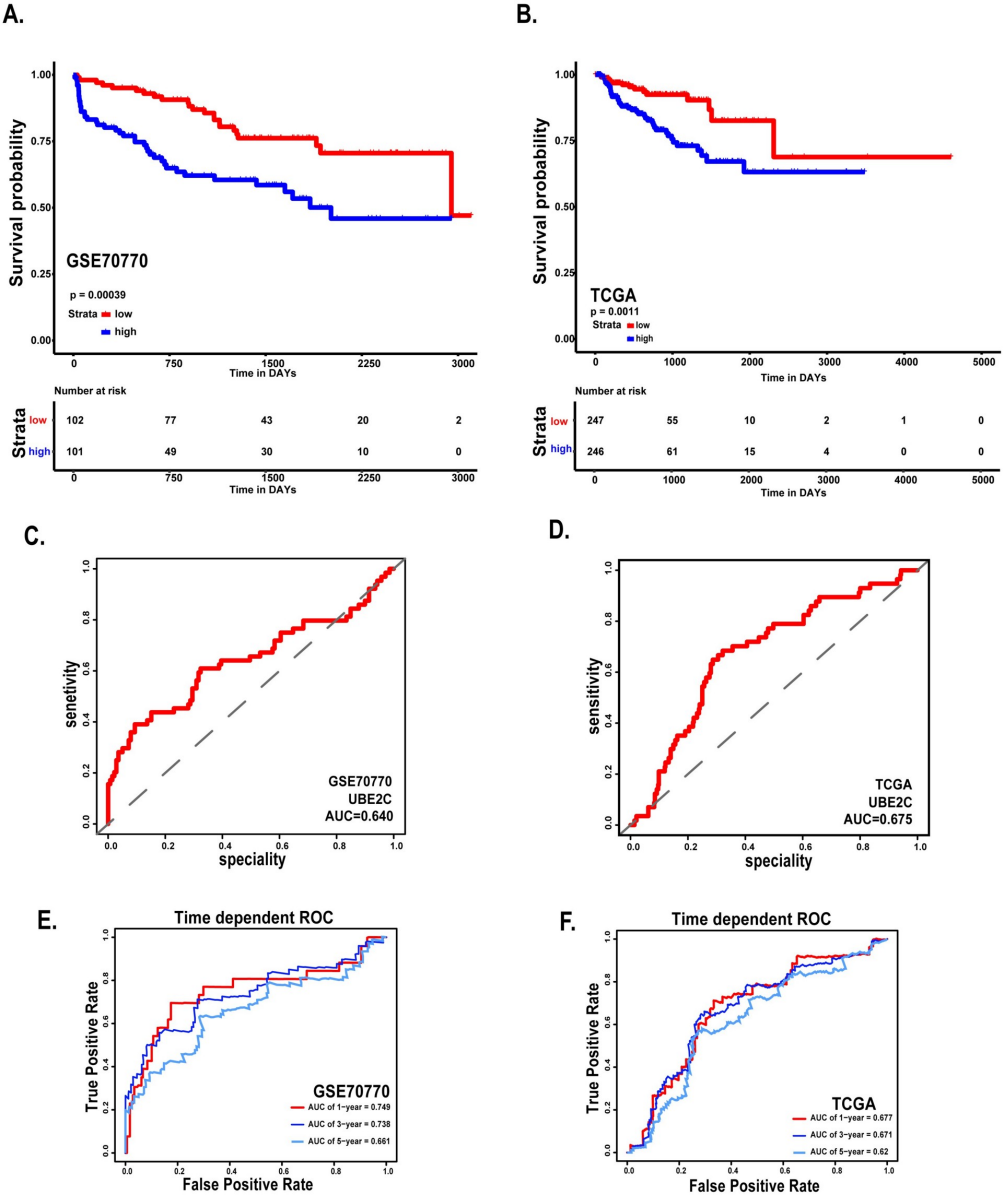
Survival analysis and ROC curve of UBE2C in GSE70770 and TCGA. (A) Disease free survival of patients based on the expression of UBE2C in GSE70770. (B) Disease free survival of patients based on the expression of UBE2C in TCGA. (C) ROC curve of UBE2C in GSE70770. (D) ROC curve of UBE2C in TCGA. (E-F) Time-dependent ROC curves. The AUCs at 1, 3, and 5 years were used to assess prognostic accuracy (left: GSE70770, right: TCGA). *: p<0.05; **: p<0.01; ***: p<0.001; ****: p<0.0001.

Considering the clinical application value of candidate hub genes, firstly, we analyzed the correlation between candidate hub genes and Gleason score of prostate cancer patients in GSE70770 (Fig 5). Secondly, we have validated the role of UBE2C in the T-stage and N-stage of prostate cancer (Fig 6). Then we performed the disease-free survival and expression analysis of UBE2C in GSE70770 (three samples was excluded because of the absence of the follow up months), GSE116918 and TCGA by KM curve and boxplot (Figs 6 and 7). The correlations between clinical parameters and the expression of UBE2C included in Table 2 was done by SPSS version 19.0. Continuous variables were analyzed by independent t tests, as for categorical variables which were analyzed by chi-square test. By performing univariate and multivariate cox regression analysis (Table 3), the correlations between clinical parameters and disease-free survival were done by R software. Rms package of R software was used to do nomogram plots analysis (S2 Fig). ROC curve analysis was done with survivalROC and pROC package (Figs 7 and S3).

**Table 2:** The relationships between UBE2C and clinical parameters (n = 203).

Clinical parameters		Low UBE2C (n = 102)	High UBE2C (n = 101)	P value
Age	Mean±Std. Deviation	60.31±6.567	60.59±6.685	0.863
	Median(Minimum ~Maximum)	62(41~72)	61(42~73)	
Gleason Score	≤6	31(30.4%)	6(5.9%)	<0.0001
	7 = 3+4	46(45.1%)	55(54.5%)	
	7 = 4+3	16(15.7%)	23(22.8%)	
	8–10	7(6.9%)	17(16.8%)	
	Unknown	2(2.0%)	0(0.0%)	
PSA	Mean±Std. Deviation	9.8117±12.04088	9.2606±5.34981	0.187
	<4	5(4.9%)	4(4.0%)	0.798
	4–10	70(68.6%)	66(65.3%)	
	>10	25(24.5%)	30(29.7%)	
Extra-capsular extension (ECE)	Yes	48(47.1%)	71(70.3%)	<0.001
	No/Unknown	54(52.9%)	30(29.7%)	
Positive surgical margins (PSM)	Yes	30(29.4%)	63(62.4%)	0.215
	No/Unknown	72(70.6%)	38(37.6%)	
Biochemical relapse (BCR)	Yes	23(22.55%)	41(40.6%)	0.006
	No/Unknown	79(77.5%)	60(59.4%)	

**Table 3:** Univariate and multivariate Cox regression analysis for prostate cancer relapse-free survival.

Variate		Univariate		multivariate	
		HR (95%CI)	P value	HR (95%CI)	P value
Age		1.011(0.942–1.084)	0.763		
UBE2C		3.142(2.218–4.449)	0.0001	2.796(1.762–4.436)	0.0001
PSA		1.022(1.005–1.038)	0.01	1.014(0.992–1.037)	0.217
Gleason	< = 6	1		1	
	7 = 3+4	6.186(1.462–26.175)	0.013	3.576(0.811–15.770)	0.092
	7 = 4+3	16.085(3.752–68.947)	<0.0001	8.974(1.984–40.601)	0.004
	8–10	32.987(7.539–144.331)	<0.0001	12.568(2.602–60.703)	0.002
ECE	YES	1		1	
	NO/unknown	0.393(0.227–0.683)	0.001	0.619(0.335–1.144)	0.126
PSM	YES	1		1	
	NO/unknown	0.455(0.278–0.744)	0.002	0.499(0.295–0.842)	0.009

ECE: Extra-capsular extension PSM: Positive surgical margins HR: Hazard ratio CI: Confidence interval.

### GSEA

**Fig 8:**
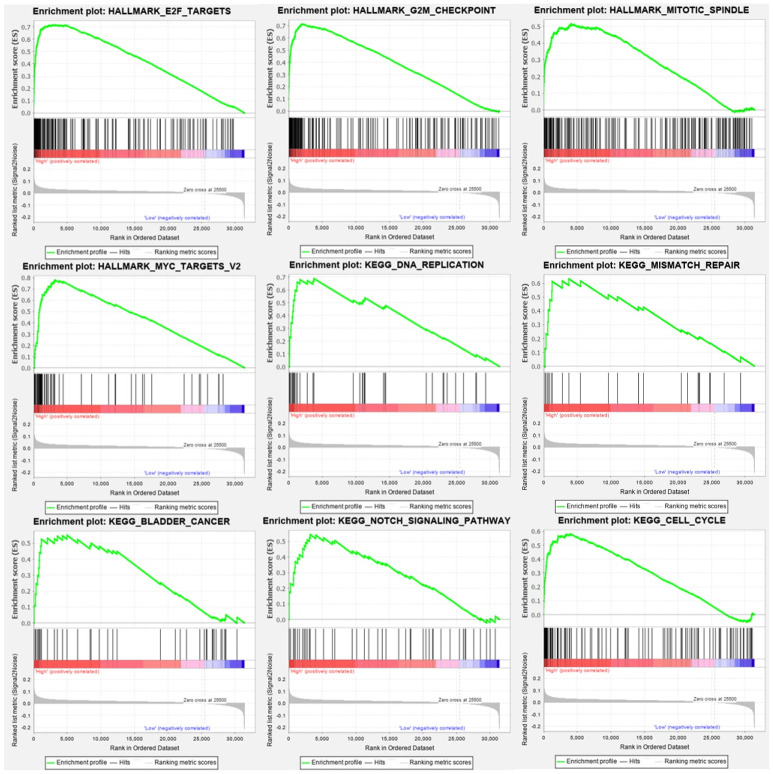
Genes enrichment analysis. The results above all meet the standards (FDR<0.25, P-value < 0.05). The results showed that high expression of UBE2C were enriched in G2M check point, cell cycle, DNA replication, E2F targets, MYC targets, Notch signaling pathway.

The GEO dataset (GSE70770) contained 206 primary prostate cancers, which were divided into two groups based on the median expression of UBE2C, high versus low. To figure out the function and effect of UBE2C, GSEA was used to do the enrichment analysis. The criteria cutoff set up as P-value < 0.05 and false discovery rate (FDR) < 0.25 (Fig 8).

## Results

### DEGs in prostate cancer

As shown in volcano plots (Fig 1A–1D), we got 527 DEGs in GSE3325, 1092 DEGs in GSE46602, 330 DEGs in GSE104749 and 1449 DEGs in GSE69223. As shown in Venn diagram (Fig 1E), a total of 243 upregulated genes and 298 downregulated genes were identified. In addition, transient receptor potential melastatin-4 (TRPM4), distal-less homeobox 1 (DLX1), collagen triple helix repeat containing 1 (CTHRC1) and asporin (ASPN) are high expression in four datasets, while K+ channel gene (KCN3), Alcohol oxidase I (AOX1), HUT11/UT-B/JK(SLC14A1), collagen type XIII alpha 1 (COL13A1), transcription elongation factor A (SII)-like 2 (TCEAL2), homeobox D10 (HOXD10), carboxypeptidase A6 (CPA6) are decreased in all datasets. (Fig 1F and 1G).

### GO and KEGG pathway analysis

To figure out the function of these DEGs, as described in S1 Table, DAVID was used to perform GO and KEGG pathway and the results were presented in S2 Table. As shown in Fig 2, biological process analysis indicated DEGs were significantly enriched in the oxidation-reduction process, negative regulation of transcription, cell-cell signaling, lipid metabolic process, cell adhesion. For the cellular component, the most significantly altered pathway was proteinaceous extracellular matrix, extracellular region. As for molecular function, cadherin binding involved in cell-cell adhesion, oxidoreductase activity was significantly enriched. KEGG pathway analysis showed that transcriptional dysregulation in cancer, TGF-beta signaling pathway, Hippo signaling pathway were essential for prostate cancer. With metascape, the results indicated that DEGs were obviously correlated with the development of prostate neoplasms (Fig 2E and 2F).

### PPI network construction and hub genes validation

In order to screen the hub genes, the different expression genes were uploaded to STRING, then PPI network was further constructed by Cytoscape (Fig 3). In order to identify the hub genes of prostate cancer, DEGs were calculated by Maximal Clique Centrality (MCC) [26], Degree method, Density of Maximum Neighborhood Component (DNMC) [22] to screen the top ten genes. The 7 genes, UBE2C, PBK, CCNB1, CDKN3, TOP2A, AURKA and MKI67, presenting in at least two methods, were recognized as candidate hub genes. But only UBE2C, screening in all methods, was recognized as of higher worth. In order to verify the role and function of 7 genes in the progression and prognosis of prostate cancer, expression level and survival analysis was performed by GEPIA (Fig 4). The results showed that 7 genes were all associated with prostate cancer prognosis, but only UBE2C, TOP2A and CCNB1 were high expression in prostate cancer samples than normal prostate gland samples, the expression of PBK, CDKN3, AURKA, MKI67 were not.

### Clinical information of the hub gene

Considering the clinical value of 7 genes, we analyzed the correlation between candidate hub genes and Gleason score of prostate cancer. As the results showed that, candidate hub genes, in particular, UBE2C was positive related with Gleason score in GSE70770 (Figs 5 and 6B). The higher expression level of UBE2C indicated the worse Gleason score. Moreover, the expression level of UBE2C increased as T-stage and N-stage of prostate cancer improved (Fig 6C and 6D). According to the validation results, UBE2C, which was highly expressed in prostate cancer (Fig 6A), in particular castration-resistant prostate cancer. And the results showed that UBE2C was essential for predicting the disease-free survival of prostate cancer and may play an important role in prostate cancer (Figs 7 and S4). UBE2C was confirmed as the hub gene of prostate cancer. As shown in Table 2, based on the median expression of UBE2C, primary prostate cancer samples in GSE70770 were divided into low versus high group. As we expected, extra-capsular extension (ECE), Gleason score and biochemical relapse (BCR) had remarkable differences between the two groups, while no significant differences were observed in age, PSA and positive surgical margins (PSM). To figure out whether UBE2C could function as the independent prognostic factor of prostate cancer, the effect of clinical phenotype on relapse-free survival was presented by Cox regression (Table 3). According to the results, UBE2C functioned as a risk factor in prostate cancer progression (HR 2.796; 95%CI 1.762–4.436; P<0.0001). ROC curve was applied to present the predictive accuracy of UBE2C (AUC = 0.64 in GSE70770, AUC = 0.675 in TCGA) in DFS analysis (Fig 7). In GSE70770, the AUC of prostate cancer BCR at 1-year is 0.749, 0.738 for 3-year, and 0.661 for 5-year. And the results in TCGA (1-year AUC: 0.677, 3-year AUC: 0.671, 5-year AUC: 0.62) were similar to GSE70770. UBE2C combined with clinical features achieved a more remarkable area under the curve of ROC which was significantly superior to UBE2C alone (S3 Fig). Using nomogram that integrated clinicopathological features (S5 Fig), we predict survival probabilities of patients to improve the patients’ living condition. For localized prostate cancer, radical prostatectomy combined with radiation has become the first choice, although it can bring some adverse effects to patients. Previous study has showed UBE2C was highly correlated with chemoresistance and radiotherapy resistance of prostate cancer [27–30]. According to our results, UBE2C highly expressed in prostate cancer, especially in castration-resistant prostate cancer and UBE2C was correlated with the neuroendocrine prostate cancer biomarkers, such as RB1 and LDHA (S4 Fig). It indicated that targeting UBE2C may provide new therapy idea for prostate cancer, especially for castration-resistant prostate cancer.

### GSEA

As shown in Fig 8, high expression of UBE2C were positive with G2M check point, cell cycle, DNA replication. Furthermore, E2F targets, MYC targets, Notch signaling pathway were also significantly enriched companying with UBE2C expression. As we expected, UBE2C is positively correlated with bladder cancer progression as well as renal cell carcinoma, pancreatic cancer.

## Discussion

Ubiquitin, which was a conserved protein containing 76 amino acids, could be included in the process of post-translation for protein degradation [31]. We confirmed that ubiquitin could be conjugated to lysine residue of target protein through multi-step reactions of enzymes [31–33]. Ubiquitin-activating enzyme (E1), ubiquitin-conjugating enzyme (E2) and ubiquitin ligases (E3) were all involved in the ubiquitination. As we all know, ubiquitination, which was one of the post-translational modifications, began with activation of ubiquitin molecule and was terminated by the linkage of ubiquitin to the target protein [32,33]. Ubiquitination was essential for the regulation of a number of cellular processes among almost all mammalian cells [34,35]. The modification involved in the progression of many severe diseases, like infection, inflammation response, cancer, neurodegeneration [34–36]. It provided us with a novel strategy for the therapeutic intervention of tumor by regulating the activity of ubiquitin enzyme [35].

Based on current study, UBE2C, which could not only drive prostate neoplasms progression but also could predict the prognosis of prostate cancer, was identified as the hub gene of prostate cancer. We have confirmed that the level of UBE2C was paralleled with the Gleason score of prostate cancer, early biochemical recurrence and poor clinical outcomes. As the results showed, the higher UBE2C level was, the worse Gleason score would be. UBE2C, which belongs to the family of E2 ubiquitin-conjugating enzyme, has played a crucial role in inducing protein degradation through ubiquitin-proteasome proteolytic (UPP) pathway in cooperation with anaphase promoting complex (APC) [37,38]. UBE2C was highly expressed in diverse tumors when compared with respective normal tissue [38,39], such as breast cancer, lung cancer, colon cancer, liver cancer, thyroid cancer, prostate cancer [40]. The mechanisms of UBE2C in promoting and regulating the development of prostate cancer deserve to be explored. And there have been several researches focused on the mechanism of UBE2C in prostate cancer. Other research has confirmed the level of UBE2C, which could be regulated by miR-381-3p, was positive correlated with the proliferation of prostate cancer cells [39]. Post-translational modification of Mediator 1(MED1) T1032 phosphorylation, which was regulated by PI3K/AKT signaling pathway [41], promoted the expression of UBE2C in prostate cancer and enhanced the role of UBE2C in promoting the proliferation of prostate cancer and in driving prostate cancer progression. Conspicuously, in PC-3 cells, UBE2C could recruit FOXA1 to its enhancers, that was mostly likely correlated with the expression of UBE2C [41] and promoted the progression of castration-resistant prostate cancer. Other research has reported that FOXM1, E2F1 and RAD51 could bind to the enhancer regions and promoter regions of UBE2C in order to regulate the expression of UBE2C [40]. Apparently, the level of UBE2C showed strong relationship with the differentiation and progression of prostate cancer and conspicuously correlated with the prognosis. Compared with low risk androgen-dependent prostate carcinoma (ADPC), UBE2C prominently upregulated in the fatal castration-resistant prostate cancer (CRPC) [37]. ADT has been considered as the first-line treatment for advanced prostate cancer. Although in a few months later, prostate cancer would progress to CRPC which had no response to the ADT [42]. The pathogenesis of CRPC was most closely to AR mutation, AR amplification, the presence of AR-V7 and abnormal activation of androgen receptors downstream signals [43]. Compared with full-length AR(AR-FL), the level of UBE2C was accurately correlated with the expression of AR variant 7(AR-V7), which could regulate the expression of UBE2C [42]. It revealed that UBE2C was the downstream target of AR-V7, which would give us a brand-new way to explore the therapy for CRPC by targeting the activity of UBE2C.

Through comprehensive bioinformatics analysis, we concluded that UBE2C could drive the progression of prostate cancer, but the analysis in this paper still had some certain limitations. First, the role of UBE2C in prostate cancer has not been proven in vivo or in vitro. It is essential for us to verify the importance of UBE2C in prostate cancer and explore the mechanism of UBE2C in regulating the development of prostate cancer by experimental approach. Second, the study of chemotherapy and radiotherapy resistance of prostate cancer patients is very important. It is of great worth to study the relationship between the expression of UBE2C and the treatment resistance of prostate cancer. So we should pay attention to performing clinical trials and we also should focus on the prevention, the treatment and the care of cancer.

## Supporting information

S1 Fig The flowchart of inclusion criteria of GEO gene expression profiles. (DOCX)

S2 Fig DEGs in GSE104749, GSE3325, GSE69223, GSE46602 and pathway analysis. (A-D) Heatmap plots of four GEO databases. Red plots symbolized upregulation genes, green represented downregulation genes, the black plots represented the genes with no significant expression change. (A) GSE3325 (B) GSE69223 (C) GSE104749 (D) GSE46602. (E) The barplot of GO and KEGG pathway analysis of DEGs.
(DOCX)

S3 Fig The ROC curve of UBE2C combined with clinicopathological phenotype in GSE70770. (A) UBE2C and Gleason score (AUC = 0.7475). (B) UBE2C, Gleason score and age (AUC = 0.8202). (C) UBE2C, Gleason score, age and T-stage (AUC = 0.8459). (D) UBE2C and MKI67 (AUC = 0.726) (E) UBE2C and CDKN3 (AUC = 0.677). (F) UBE2C and CCNB1 (AUC = 0.674). (G) UBE2C and TOP2A (AUC = 0.649). (H) UBE2C and PBK (AUC = 0.645). (I). UBE2C and AURKA (AUC = 0.659).
(DOCX)

S4 Fig The role of UBE2C in castration-resistant prostate cancer. (A) DFS in GSE116918. (B) ROC curve of UBE2C in GSE116918. (C) Time dependent ROC of UBE2C in GSE116918. (D) The correlation of UBE2C and RB1 in castration-resistant prostate cancer. (E) The correlation of UBE2C and LDHA in castration-resistant prostate cancer.
(DOCX)

S5 Fig Nomogram of prostate cancer to predict 3-year, 5-year survival probability. (A) Nomogram of prostate cancer. B-C: Nomogram’s calibration chart. The dotted line represents an ideal reference line where the predicted probability matches the proportion of observations. B: Nomogram-Predicted Probability of 3-year survival. C: Nomogram-Predicted Probability of 5-year survival.
(DOCX)

S1 Table Identification of DEGs in prostate cancer among four databases. (DOCX)

S2 Table GO and KEGG signaling pathways (TOP 10). (DOCX)
