# Supplementary figures and images for: Identification of UBE2C as hub gene in driving prostate cancer by integrated bioinformatics analysis
Source: PLoS One. 2021 Feb 25;16(2):e0247827. doi: 10.1371/journal.pone.0247827 (PMC7906463; doi:10.1371/journal.pone.0247827)

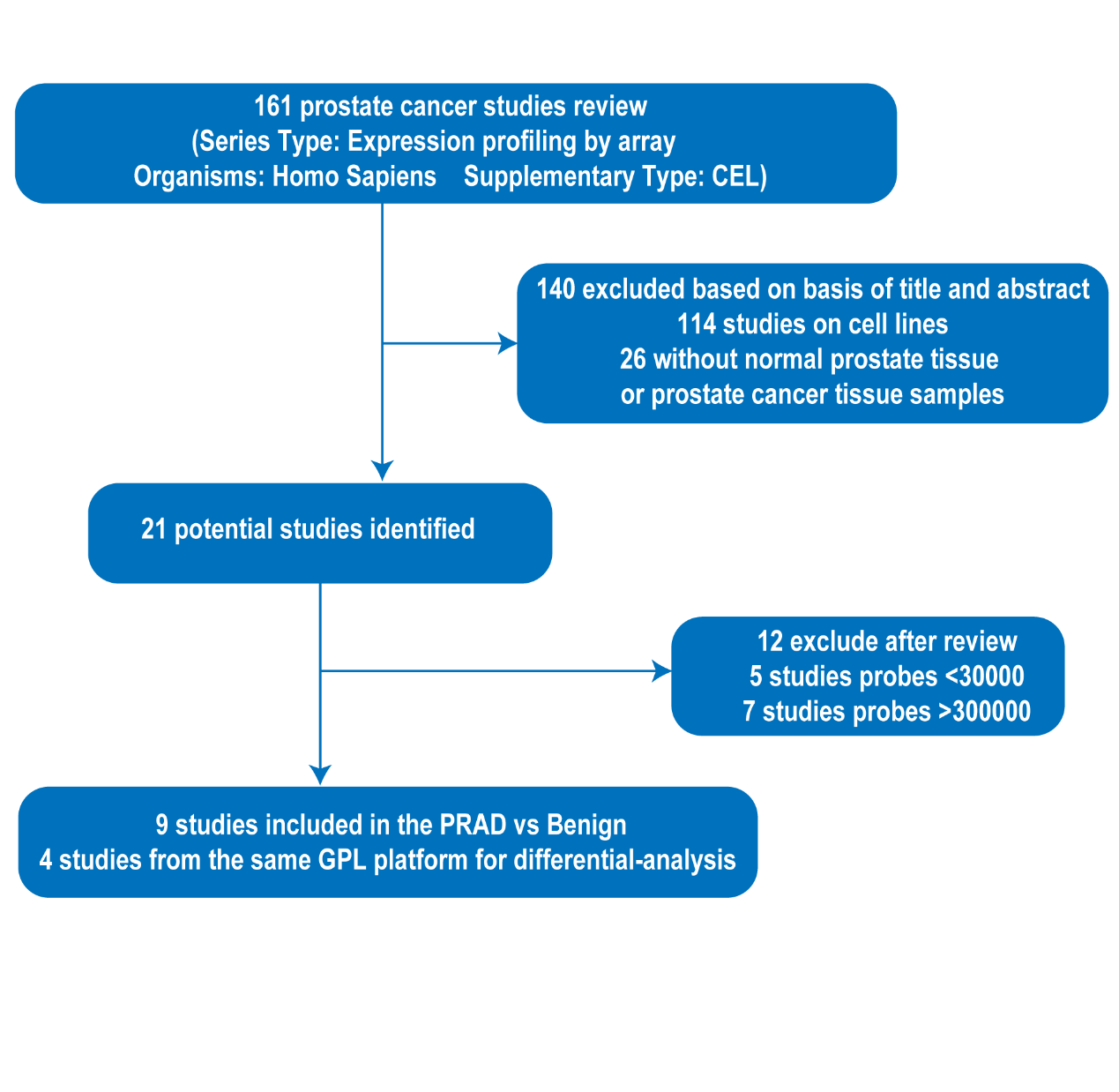


**S1 Fig. The flowchart of inclusion criteria of GEO gene expression profiles.**

Supplement: S1 Fig — (DOCX) [file pone.0247827.s001.docx]
