# Supplementary material for: Identification of UBE2C as hub gene in driving prostate cancer by integrated bioinformatics analysis
Source: PLoS One. 2021 Feb 25;16(2):e0247827. doi: 10.1371/journal.pone.0247827 (PMC7906463; doi:10.1371/journal.pone.0247827)
Supplement: S2 Fig — (A-D) Heatmap plots of four GEO databases. Red plots symbolized upregulation genes, green represented downregulation genes, the black plots represented the genes with no significant expression change. (A) GSE3325 (B) GSE69223 (C) GSE104749 (D) GSE46602. (E) The barplot of GO and KEGG pathway analysis of DEGs. (DOCX) [file pone.0247827.s002.docx]

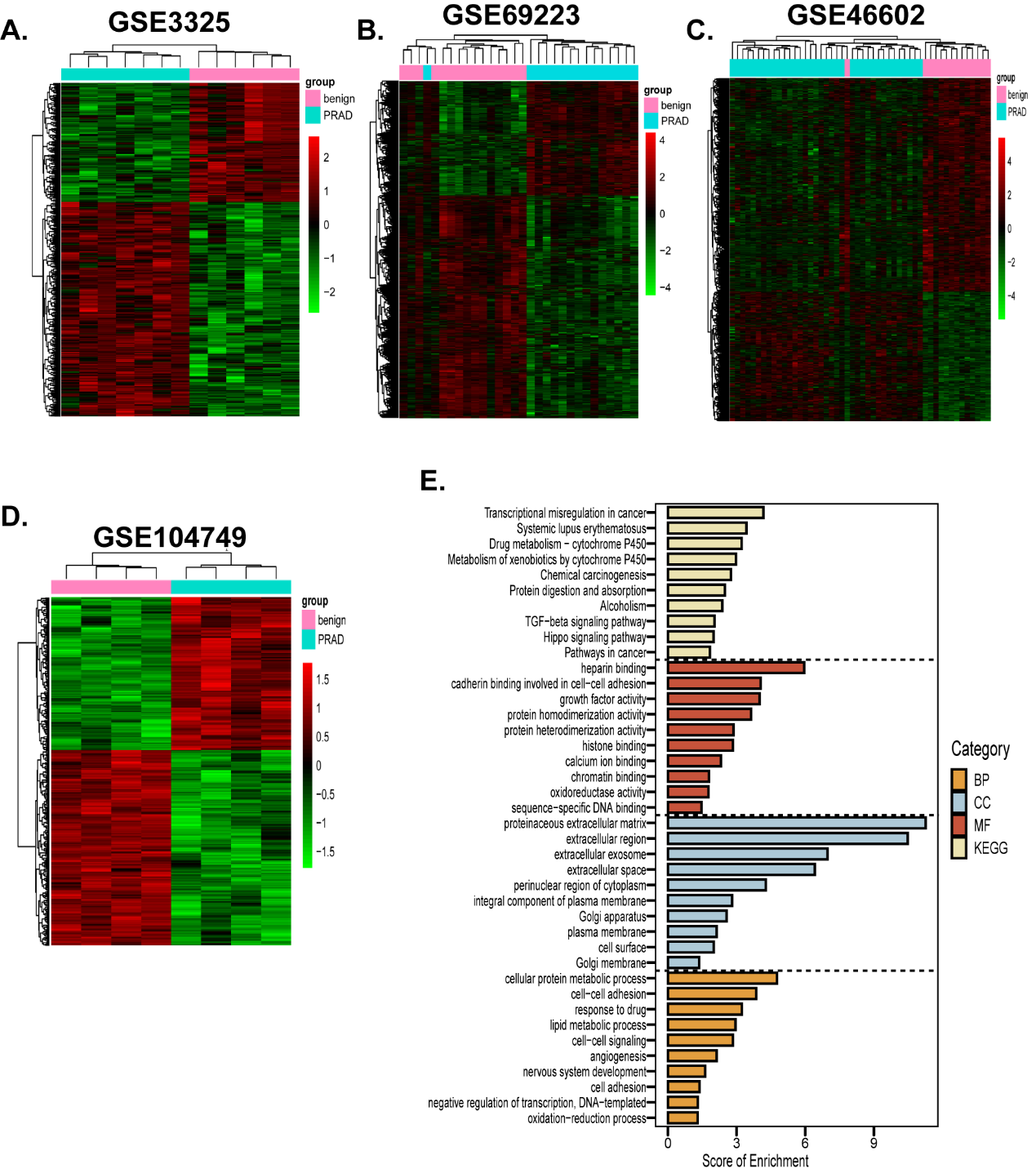


**S2 Fig. DEGs in GSE104749, GSE3325, GSE69223, GSE46602 and pathway analysis**

(A-D) Heatmap plots of four GEO databases. Red plots symbolized upregulation genes, green represented downregulation genes, the black plots represented the genes with no significant expression change. (A) GSE3325 (B) GSE69223 (C) GSE104749 (D) GSE46602. (E) The barplot of GO and KEGG pathway analysis of DEGs.
