# Supplementary material for: Identification of UBE2C as hub gene in driving prostate cancer by integrated bioinformatics analysis
Source: PLoS One. 2021 Feb 25;16(2):e0247827. doi: 10.1371/journal.pone.0247827 (PMC7906463; doi:10.1371/journal.pone.0247827)
Supplement: S3 Fig — (A) UBE2C and Gleason score (AUC = 0.7475). (B) UBE2C, Gleason score and age (AUC = 0.8202). (C) UBE2C, Gleason score, age and T-stage (AUC = 0.8459). (D) UBE2C and MKI67 (AUC = 0.726) (E) UBE2C and CDKN3 (AUC = 0.677). (F) UBE2C and CCNB1 (AUC = 0.674). (G) UBE2C and TOP2A (AUC = 0.649). (H) UBE2C and PBK (AUC = 0.645). (I). UBE2C and AURKA (AUC = 0.659). (DOCX) [file pone.0247827.s003.docx]

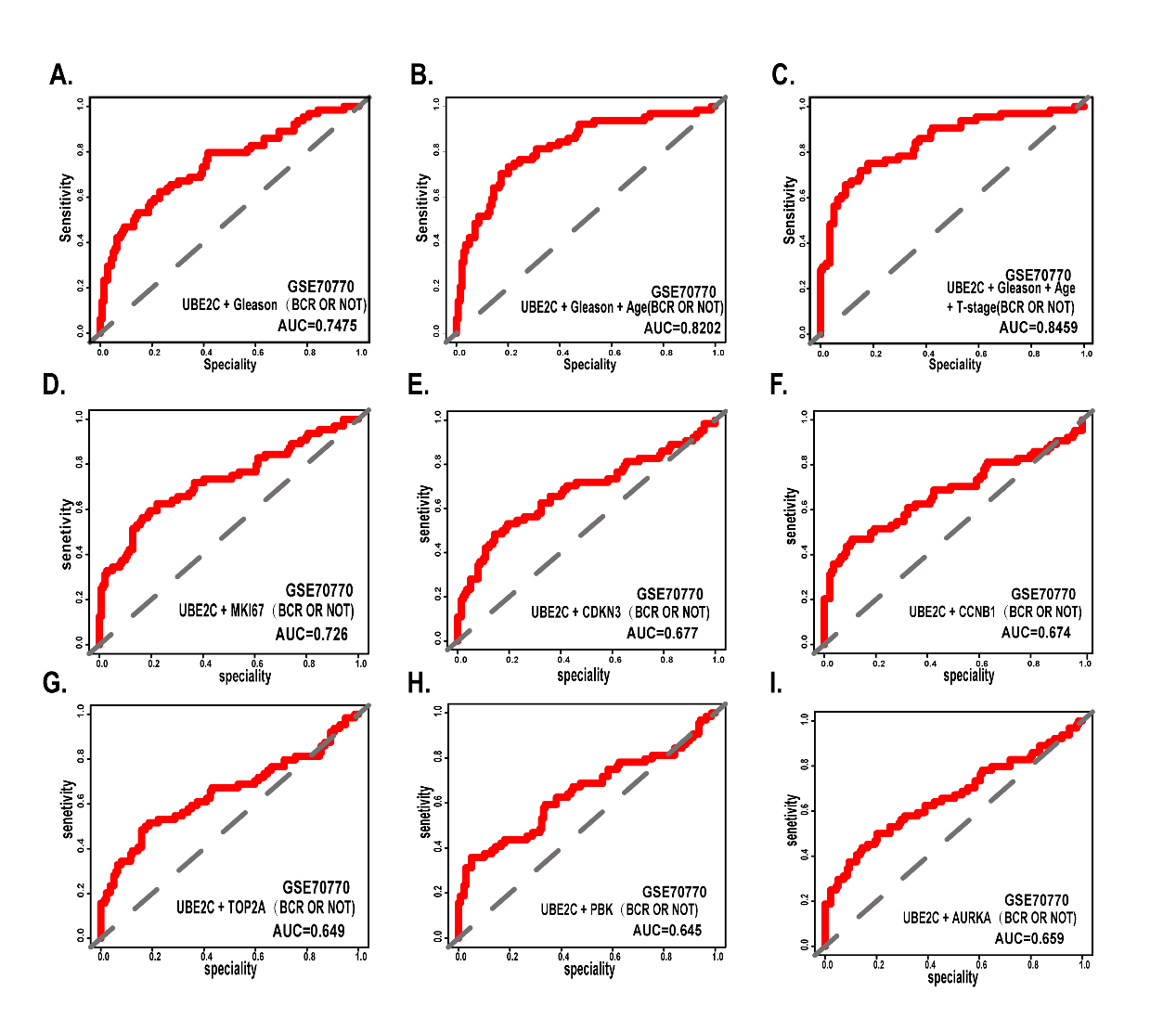


**S3 Fig. The ROC curve of UBE2C combined of clinicopathological phenotype in GSE70770.**

(A) UBE2C and Gleason score (AUC=0.7475). (B) UBE2C, Gleason score and age (AUC=0.8202). (C) UBE2C, Gleason score, age and T-stage (AUC=0.8459). (D) UBE2C and MKI67 (AUC=0.726) (E) UBE2C and CDKN3 (AUC=0.677). (F) UBE2C and CCNB1 (AUC=0.674). (G) UBE2C and TOP2A (AUC=0.649). (H) UBE2C and PBK (AUC=0.645). (I). UBE2C and AURKA (AUC=0.659).
