# Supplementary material for: Identification of UBE2C as hub gene in driving prostate cancer by integrated bioinformatics analysis
Source: PLoS One. 2021 Feb 25;16(2):e0247827. doi: 10.1371/journal.pone.0247827 (PMC7906463; doi:10.1371/journal.pone.0247827)
Supplement: S4 Fig — (A) DFS in GSE116918. (B) ROC curve of UBE2C in GSE116918. (C) Time dependent ROC of UBE2C in GSE116918. (D) The correlation of UBE2C and RB1 in castration-resistant prostate cancer. (E) The correlation of UBE2C and LDHA in castration-resistant prostate cancer. (DOCX) [file pone.0247827.s004.docx]

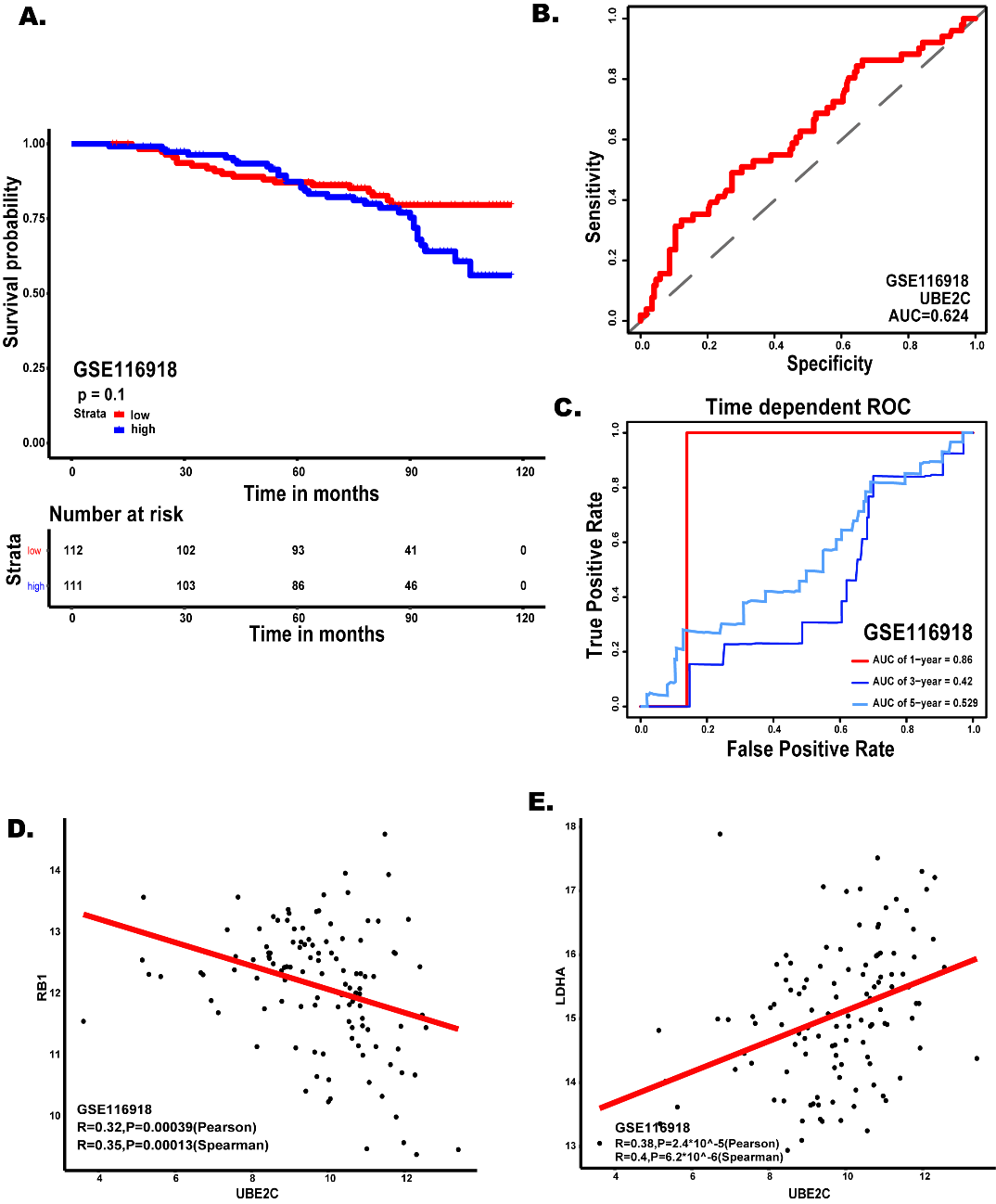


**S4 Fig. The role of UBE2C in castration-resistant prostate cancer.**

(A) DFS in GSE116918. (B) ROC curve of UBE2C in GSE116918. (C) Time dependent ROC of UBE2C in GSE116918. (D) The correlation of UBE2C and RB1 in castration-resistant prostate cancer. (E) The correlation of UBE2C and LDHA in castration-resistant prostate cancer.
