# Supplementary material for: Identification of UBE2C as hub gene in driving prostate cancer by integrated bioinformatics analysis
Source: PLoS One. 2021 Feb 25;16(2):e0247827. doi: 10.1371/journal.pone.0247827 (PMC7906463; doi:10.1371/journal.pone.0247827)
Supplement: S5 Fig — (A) Nomogram of prostate cancer. B-C: Nomogram’s calibration chart. The dotted line represents an ideal reference line where the predicted probability matches the proportion of observations. B: Nomogram-Predicted Probability of 3-year survival. C: Nomogram-Predicted Probability of 5-year survival. (DOCX) [file pone.0247827.s005.docx]

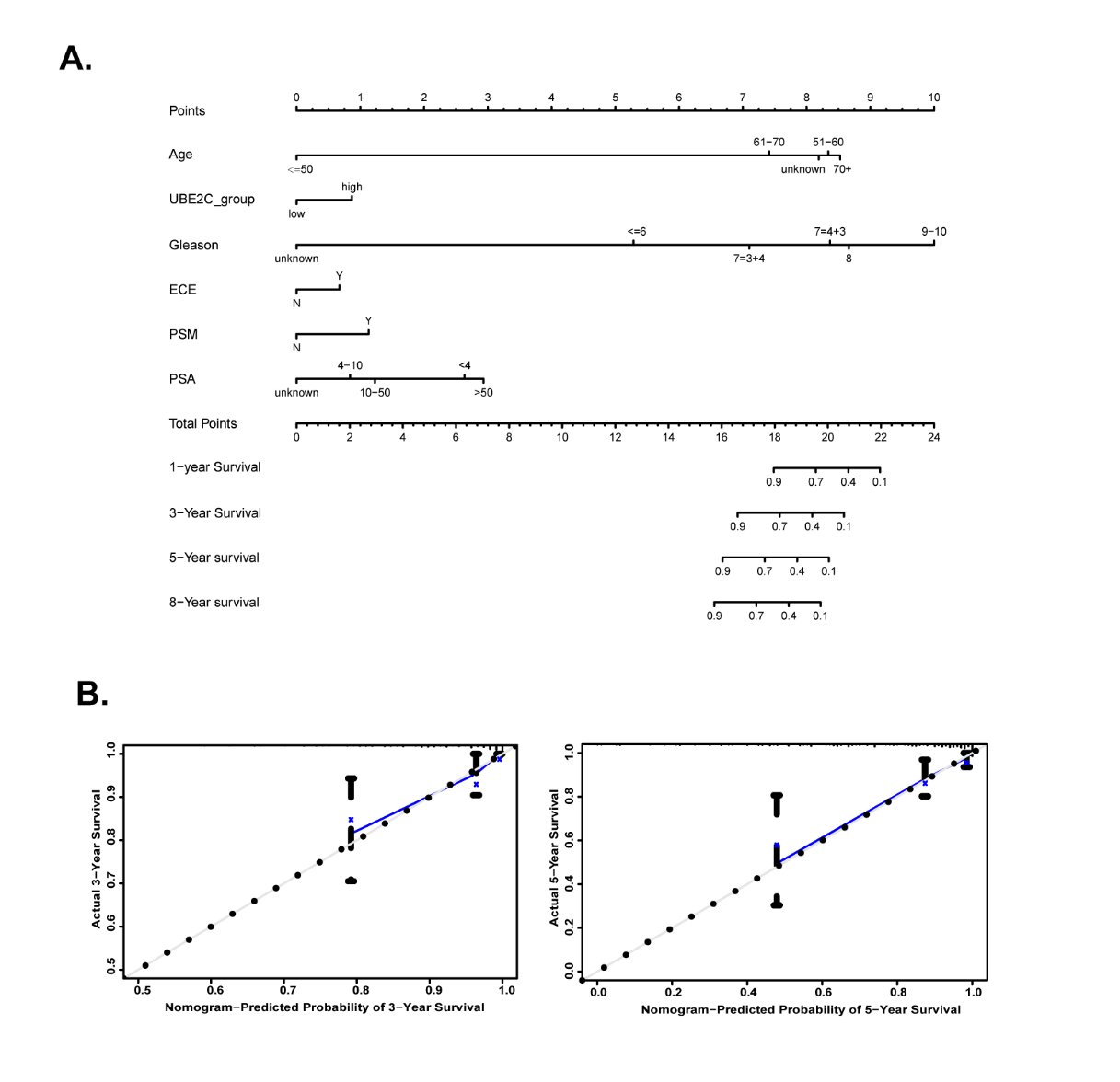


**S5 Fig. Nomogram of prostate cancer to predict 3-year, 5-year survival probability.**

(A) Nomogram of prostate cancer. B-C: Nomogram's calibration chart. The dotted line represents an ideal reference line where the predicted probability matches the proportion of observations. B: Nomogram-Predicted Probability of 3-year survival. C: Nomogram-Predicted Probability of 5-year survival.
