# Supplementary material for: Identification of UBE2C as hub gene in driving prostate cancer by integrated bioinformatics analysis
Source: PLoS One. 2021 Feb 25;16(2):e0247827. doi: 10.1371/journal.pone.0247827 (PMC7906463; doi:10.1371/journal.pone.0247827)
Supplement: S1 Table — (DOCX) [file pone.0247827.s006.docx]

**S1 Table. Identification of DEGs in prostate cancer among four databases**

| **Alternations** | **Genes** |
| --- | --- |
| **Up- regulation** | TRPM4, DLX1, CTHRC1, ASPN, THBS4, B3GAT1, PENK,DNAH5,SLC43A1, C19orf48, MARCKSL1, LOC101927870, GCNT1, LINC00992, GDF15, SIM2, GOLM1, SMIM22, APOF, TSPAN1, TMTC4, ABCC4, MS4A8, EFCAB4A, EPCAM, GLYATL1, TMEM178A, PCA3, STX19, ADRB1, COL9A2, RAP1GAP2, LOC100507472, LUZP2, BEND4, UAP1, GJB1, MYO6, HOXC6, FAM222A, AMACR, LOC101060264, PPM1E, HIST1H3A, INSM1, HPN, PCAT18, RPL22L1, C1QTNF3, ST6GALNAC1, HOXC4, CHRM3, TRIB1, NEK5, RAB17, ELL3, TBX1, RRM2, CCNB1, MICAL2, NUSAP1, AURKA, ECT2, ITGBL1, MYC, TWIST1, REPS2, EZH2, BICD1, LOC101930578, SMOC2, PCDHGA4, CXCL14, ACSM1, YPEL1, BIRC5, MKI67, C7orf13, CAMKK2, C4A, SPIN4, PCTP, GREM1, EIF4EBP1, TGM3, SNHG3, CYP2J2, APOC1, LOC101927391, RET, MEOX1, RALGAPA2, SBK1, ERG, TDRD1, KCNG3, NETO2, KCNH8, EBF2, CCNB2, ELAVL2, COL28A1, MAP2K6, FOXD1, LDLRAD3, FAM84A, LOC101927482, PLA1A, IQGAP2, TMSB15A, OR51E2, PVRL3, LFNG, MB, EGF,SFTPA1, CNTD1, COLEC12, FAM84A, C1orf53, NKAIN1, ATP7B, FBP1, SLC6A14, PYCR1, LOC286189, LOC283177, RNF157, VSTM2L, ITPR3, GAL, GREM2, KLK15, MNX1, SLC38A11, POPDC3, CNKSR2, ERVH48-1, SH3RF1, FLJ40288, HIST3H2A, MMP26, PTPRT, C1orf85, GLOD5, FGF14, TMEM132A, KIF20A, KIAA0101, COL10A1, HMMR, TOX3, UBE2C, TFF3, CDKN3, ST14, ANKRD20A12P, F2R, PPP1R14B, NUP210, PDLIM5, ARHGEF26, MIR3658, SPDEF, TK1, CGREF1, LOC101928717, C2orf72, FAM83H, FZD8, PLEKHB1, PBK, MYRIP, SLC12A8, ENTPD5, RAP1GAP, TARP, AP1S3, TRIB3,FLJ20021, CORO1B, TSPAN13, MAL2, SLC22A23, AGR2, CLDN3, TRIM36, HIST1H3E, ESRP2, PTPRN2, CHMP4C, GPR160, SLC27A2, KIAA1244, C9orf152,TRPM8, CYP39A1, PABPC1L2A, DUS1L, LOC145837, LOC100996425, CTB-167B5.2, ZNF613, EPN3,TMC5, GMDS, CLDN7, RGS17, SLC7A11, PRAC1, ALDH3B2, LOC100505938, PRR15L, TM7SF2, TLCD1, SAMD5, ZNF577, GDPD1, FASN, GALNT3, STEAP4, GJB2, ZIC2, TBC1D30, SEL1L3, LOC100996455, CADPS, SULF1 COL2A1, POSTN, NCAPG, INHBA, TOP2A, CENPF, DLX2, RUVBL1, MAFG-AS1,ANLN, COMP, LOC642852, CENPM, VCAN |
| **Down- regulation** | KCNK3, AOX1, SLC14A1, COL13A1, TCEAL2, HOXD10, CPA6, SEMA6D, PCDH9, SAMD12, UNC5B, PGR, PPARGC1A, FBXO17, ID4, NTNG2, PGM5-AS1, PRIMA1, HOXD11, ETV5, LINC00844, GPD1L, SPRY4-IT1,SERPINB11, ALAD, CFC1, TRIM29, NBL1, FOXQ1, MSMO1, CRISPLD2, DMKN, FHDC1, CSTA, GPRC5B, CAPG, BEX1, MME, FADS1, DUOX1, NDRG2, GAS1,DSC3, CYP4B1, UPK1A, AFAP1L2, BCL11A, ALOX12P2, APCDD1, LOC101928635, LINC00086, TMEM246, MALL, ST3GAL5, GPR126, GATA3, BASP1, RBP4, COL9A1, GMNN, PCAT4, SCGB1A1, SDC1, ADAMTS1,DUOXA1, HOXD13, APOBEC3C, LOC100506119, FBLN2, CXorf57, ANKRD35, EDNRB, GPC6, C2orf40, PPARG, MLC1, KCNJ3, SBSPON, ACOT11, RCAN2, TMEM132C, PTGFR, ANKDD1A, KIF7, UCHL1, CABP1, NPY6R, KRT222, CLIP2, IQCA1 GOLT1A, ADAMTSL3, RHOBTB1, DUSP5P1, ID1,PRKG1, SCGB3A1, ASPA, JAKMIP1, SLC39A2, PRRT2, TGM4, WDR7, CLSTN2, AQP3, LOC100507351, SLC52A3, C10orf82, VSIG2, SOWAHA, SYT16, DUSP2, LOC642426, TIMP4, PHYHIPL, CYP3A5, ANGPT1, CCDC8, COL4A6, PTGDS, TCF7L1, NUDT10, ADAMTS9-AS2,COL17A1, KRT23, VWA5A, TP63, JUP, KRT14, AJUBA, HLF, WIF1, SNAI2, TMLHE, FAM83B, LOC101929880, ZNF483, CAV2, L3MBTL4, KANK4, CRYAB, SYNGR1, PARM1, MEIS2, FAM107A, LGR6, GJA1, HSPB6, ACAA2, SMOC1, C8orf88, CFD, GSTM1,C11orf45, SHROOM4, SPATA6,ZEB2, MCC, LOC100653086, DENND2A, LOC102723845, TGFB3, STOM, EDN3, DPT, TGFBR3, ZNF711, FRMD6, UBXN10-AS1, FEZ1, COL27A1, MEG3, SKIDA1, BEND5, TSHZ3, BST2, SRD5A2, GPM6B, ARHGAP22, KCNJ8, TSPAN18, PTN, GBP2, SMARCD3, SERPINF1, PALD1, RND3, LPAR1, GBP1, ANO5, STOX2, CES1,DNAJB4, CACHD1, LARGE, RNF180, LOC728061, CPXM1, FXYD6, ANP32E, PPP1R3C, SCN7A, EFEMP1, SLITRK6, NARR, FAM20A, S100A6, CLIP4, EHD2, S100A4, SCD5, EPHA5, RHOJ, HEY1, NDNF, S1PR3, ACSS3, CXCL12, PNMAL1, IGF1, ITIH5, BDH2, HIF3A, LINC00537, RGN, AGPAT4, TGFB2, RP11-710C12.1, PEG3-AS1, IFI16, NELL2, SPOCK3, CLU, CFH, ATRNL1, GSTM3, HSD11B1, PTGS1, SCARA3, FZD7, SNX7, LDB3, VSTM4, PCDH18, PRKCA, CLIC, PLCL1, GSTM5, KITLG, SLIT2, CECR6, GSTM4, PAPPA, OGN, EPHA7, NRK, ARMCX1, SLC16A5, NEFH, CPAMD8, CGB7, EFS, BNIPL, SLC22A17, MINOS1-NBL1, ANG, GSTM2, PDE8B, KLF8, FADS2, ANKRD65, LOC100289090, ST6GALNAC2, AL833181, CPM, KCTD14, SH3BGRL2, CDC42EP4, TTC22, SLC18A2, AMT, DPYS, GPR56, CD38, RMST, STXBP5L, MUC3B, SRPX, GSTO2, CCDC178, ANO4, ST8SIA1, C2orf88, AP1G2, IQSEC3, GATM, LOC158434 |
