# Supplementary material for: Identification of UBE2C as hub gene in driving prostate cancer by integrated bioinformatics analysis
Source: PLoS One. 2021 Feb 25;16(2):e0247827. doi: 10.1371/journal.pone.0247827 (PMC7906463; doi:10.1371/journal.pone.0247827)
Supplement: S2 Table — (DOCX) [file pone.0247827.s007.docx]

**S2 Table: GO and KEGG signaling pathways (TOP 10)**

| Term | Pathway | PValue |
| --- | --- | --- |
| **GO-BP** |  |  |
| GO:0055114 | oxidation-reduction process | 0.049663 |
| GO:0045892 | negative regulation of transcription, DNA-templated | 0.049164 |
| GO:0098609 | cell-cell adhesion | 0.000139 |
| GO:0042493 | response to drug | 0.000591 |
| GO:0007155 | cell adhesion | 0.041779 |
| GO:0007267 | cell-cell signaling | 0.00144 |
| GO:0007399 | nervous system development | 0.023365 |
| GO:0044267 | cellular protein metabolic process | 1.71E-05 |
| GO:0001525 | angiogenesis | 0.00736 |
| GO:0006629 | lipid metabolic process | 0.001106 |
| **GO-CC** |  |  |
| GO:0005578 | proteinaceous extracellular matrix | 5.48E-12 |
| GO:0005886 | plasma membrane | 0.007373 |
| GO:0070062 | extracellular exosome | 1.06E-07 |
| GO:0005576 | extracellular region | 3.34E-11 |
| GO:0005615 | extracellular space | 3.80E-07 |
| GO:0005887 | integral component of plasma membrane | 0.001578 |
| GO:0005794 | Golgi apparatus | 0.00273 |
| GO:0048471 | perinuclear region of cytoplasm | 5.25E-05 |
| GO:0009986 | cell surface | 0.009963 |
| GO:0000139 | Golgi membrane | 0.043538 |
| **GO-MF** |  |  |
| GO:0042803 | protein homodimerization activity | 0.000232 |
| GO:0005509 | calcium ion binding | 0.0048 |
| GO:0046982 | protein heterodimerization activity | 0.001362 |
| GO:0043565 | sequence-specific DNA binding | 0.033915 |
| GO:0098641 | cadherin binding involved in cell-cell adhesion | 8.86E-05 |
| GO:0003682 | chromatin binding | 0.015984 |
| GO:0008201 | heparin binding | 1.11E-06 |
| GO:0008083 | growth factor activity | 9.87E-05 |
| GO:0016491 | oxidoreductase activity | 0.017 |
| GO:0042393 | histone binding | 0.001433 |
| **KEGG** |  |  |
| hsa05202 | Transcriptional misregulation in cancer | 6.70E-05 |
| hsa05200 | Pathways in cancer | 0.014637 |
| hsa05322 | Systemic lupus erythematosus | 0.000373 |
| hsa05034 | Alcoholism | 0.004163 |
| hsa04390 | Hippo signaling pathway | 0.010098 |
| hsa00982 | Drug metabolism - cytochrome P450 | 0.0006 |
| hsa00980 | Metabolism of xenobiotics by cytochrome P450 | 0.00106 |
| hsa05204 | Chemical carcinogenesis | 0.001766 |
| hsa04974 | Protein digestion and absorption | 0.003233 |
| hsa04350 | TGF-beta signaling pathway | 0.009236 |
